# Predictive roles of Asprosin and Gremlin-1 expression in Egyptian pediatric patients with type 1 diabetes

**DOI:** 10.1038/s41598-024-82214-z

**Published:** 2025-02-20

**Authors:** Al-shimaa M. Abas, Marwa M. Esawy, Fatma Kamel, Mohamed Ali

**Affiliations:** 1 Biochemistry Department, Faculty of Science, Zagazig University, PO Box- 44519, Zagazig, Egypt; 2 Clinical Pathology Department, Faculty of Human Medicine, Zagazig University, PO Box- 44519, Zagazig, Egypt; 3 Bachelor of Chemistry & Biochemistry, Faculty of Science, Zagazig University, PO Box- 44519, Zagazig, Egypt

**Keywords:** Adipokines, Asprosin, Gremlin-1, Prediction, Diabetes mellitus, Biochemistry, Biomarkers, Endocrinology

## Abstract

Diabetes mellitus type 1 (insulin-dependent) (T1DM) is characterized by the selective destruction of the insulin-producing pancreatic beta. Asprosin and Gremlin-1 may have roles in T1DM, however these roles are not fully established. The expression of Asprosin and Gremlin-1 genes and their protein levels, was considerably higher in T1DM patients’ blood compared to the control group (*P* < 0.001). The area under the curve of 50 age- and sex-matched control persons was compared to that of serum T1DM patients. Gremlin-1 and Asprosin serum quantities were measured by ELISA, and real-time PCR was used to evaluate the expression of their genes in peripheral blood. Serum Asprosin concentration, Asprosin expression, serum Gremlin-1 concentration, and Gremlin-1 expression were 0.87, 0.997, 0.912, and 0.949, respectively. The Asprosin expression was the best marker for T1DM diagnosis with 96% sensitivity, 92% specificity, and 94% accuracy. Asprosin expression can significantly increase the risk of T1DM by 276 folds, followed by Gremlin-1 expression by 84.33 folds. The best diagnostic for T1DM diagnosis was Asprosin expression, which had 96% sensitivity, 92% specificity, and 94% accuracy. The risk of type 1 diabetes can be raised by 276 times when asprosin expression is present, and by 84.33 times when Gremlin-1 expression is present.

## Introduction

One type of autoimmune disease is T1DM that develops when the body is unable to manufacture insulin^1^. A substantial body of research supports the role of early childhood food, infections, environmental variables, and other nongenetic factors in the disease’s etiology, which is linked to genetic susceptibility. T1DM incidence has remarkably steadily increased over the past 60 years, prompting increasing research into the disease phenotype^2^. Despite enormous medical progress over the past century, there is still no cure, so better prevention and treatment are crucial to halting the spread of disease in the future^3^. From one-third to half of those with diabetes suffer from organ and tissue damage due to the disease’s significant association with problems that are both microvascular and macrovascular disorders^4^.

Thus, the combination of multiple biomarkers may yield a more accurate prognosis of those who are most at risk of getting prediabetes and eventually acquiring diabetes. In their research on diabetes, experts talked about the pathogenic function of adipokines. New insights into the role of adipokines in diabetes were provided^5^. It was once believed that adipose tissue served as a long-term energy reservoir from which free fatty acids were released during fasting to meet the body’s energy requirements. The adipocytes are powerful secretory cells that can secret a large number of adipokines that regulate a variety of vital biological and physiological processes, including glucose and lipid metabolism, appetite, inflammation, immune response, and cardiovascular homeostasis. Adipokines are the name given to these secretory byproducts of adipocytes^5^. Asprosin and Gremlin-1 are two adipokines that are highly expressed in white adipose tissue (WAT) and have an impact on diabetes^5^.

A recently identified protein hormone called Asprosin is made in WAT. It is brought on by fasting and is thought to affect the liver, resulting in a rapid glucose release into the bloodstream, and triggering the synthesis of compensatory insulin^6^. For both overall survival and brain function during fasting, the liver-related glucose release into the blood circulation is essential. Since hypoglycemic episodes can result in heart arrhythmias, strokes, or other potentially fatal consequences, they are one of the major reasons for the increased mortality in T1DM^7^.

Gremlin-1 is a protein that can be found in soluble and cell-associated forms^8^. It is a bone morphogenetic protein (BMP) antagonist that belongs to the Transforming Growth Factor-β (TGF-β) superfamily and inhibits BMP signaling to precisely control BMP gradients. Gremlin-1 has lately been linked to fibrotic disorders, despite its primary involvement in organogenesis and limb patterning. Due to being brought on by high glucose levels, it was first identified as a significant contributor to diabetic kidney fibrosis; however, it is now known to also be linked to fibrosis of the skin, eyes, liver, lungs, and skin. Human Gremlin-1 mRNA expression is extensively seen in healthy organs. Additionally, it appears to be strongly expressed in specific cell types, such as fibroblasts, astrocytes, and neurons^9^.

There were numerous developments and breakthroughs in the management and treatment of diabetes. These have included advanced insulin pumps and novel drugs^10^. Also, Insulin has its limitations for achieving normal glucose levels, including the risk of hypoglycemia^11^. The crucial future of T1DM will be disease-modifying therapies have the potential to preserve β cells in the early stages of the disease, stop the autoimmune process, and regenerate β-cell activity in the clinical phase. Immunotherapy looks to be an effective disease-modifying therapy in T1DM^12^. It has been suggested that Asprosin antibodies may be a treatment option to prevent appetite, especially in metabolic syndrome and diabetes^13^. Mishra et al.^14^ demonstrated that anti-Asprosin monoclonal antibodies are dual-effect pharmacologic therapy that targets two key pillars of metabolic syndrome, overnutrition and hyperglycemia. The addition of anti–Gremlin 1 alone significantly improved insulin-stimulated glucose uptake, and this sensitizing effect was related to the initial insulin response^15^.

This study hypothesis was that genes expression of both Asprosin and Gremlin 1 may be of value in type 1 diabetes mellitus prediction. It might be subsequently correlated with clinical and metabolic changes of diabetes mellitus such as obesity, dyslipidemia, insulin resistance, and subsequent hyperinsulinemia. The purpose of this study was to investigate the roles of adipokines (Asprosin and Gremlin-1) in the prediction of T1DM patients. Additionally, investigation of the correlation between these markers and metabolic alterations in T1DM was one of the goals. The objectives of this study were identifying Asprosin and Gremlin 1 gene expression in pediatric with T1DM, to assess their potential for early T1DM prediction, and correlate these markers’ changes with the clinical, metabolic, and consequences of the disease.

## Materials and methods

### Study design

A case-control design was applied in this study. At Zagazig University Hospitals, samples from T1DM patients and healthy people had been collected by 2022–2023. Under IRB No. 9874-2-10-2022, the Faculty of Medicine Institutional Review Board approved this study, which was established in accordance with the Declaration of Helsinki. All methods were performed in accordance with relevant guidelines and regulations. Before being enrolled, every patient read and signed the study informed consent form. Patients were aware of the study purpose, risks, and benefits. Informed consent was obtained from all participants and/or their legal guardians.

### Subjects

A 95% confidence interval (CI) and a statistical power of 80% were used to calculate the sample size. An earlier study^16^ served as the source for the mean of Asprosin between cases and controls (2.74 and 2, respectively) and their standard deviations (0.6 and 1.75, respectively). The Epi Info program version 6 was used to accomplish these calculations (Atlanta, Georgia, USA). The sample size was 50 participants in each group. Regarding patient’s enrollment, fifty consecutive patients with T1DM were enrolled at the time of study.

The American Diabetes Association’s criteria are used to determine whether someone has diabetes based on their fasting glucose readings^17^. To rule out organic disease, blood tests were performed, and a medical history was gathered. The study did not include patients who were expecting, had cancer, or had an active infection. The control group included participants with normal blood glucose levels whom the sex and age were matched.

### Sampling

Everyone participant in the current study was informed about the 12-hour fast. Whole blood was obtained after an 8-hour fast in 1 plain tube (tube with no anticoagulant) and 2 EDTA tubes (**BD Vacutainer**,** Becton Dickinson & Co.**,** NJ**). We collected the plain tubes and let them 30 min to clot at room temperature before the serum was separated using a centrifuge at 1200 x g for 10 min. Serum Gremlin-1 concentrations, serum Asprosin concentration, fasting insulin, and fasting glucose were all measured in serum. The HbA1c assay was conducted using the first EDTA tube, and Gremlin-1 and Asprosin gene expression were examined using the second EDTA tube. An additional sample was collected in a plain tube after completing 12-hour fasting in order to separate serum for the lipid profile study.

### Calculation

#### Routine laboratory tests

The lipid profile, which includes low-density lipoprotein cholesterol (LDL-C), high-density lipoprotein cholesterol (HDL-C), and total cholesterol, triglycerides, was evaluated in the lab using a Modular Analyzer Cobas 8000-C702 (**Roche**,**Germany**). Fasting glucose levels were assessed on a Modular Analyzer Cobas 8000-C702 (**Roche**,**Germany**). The HbA1C test was performed using the Modular Analyzer Cobas 6000-C501 (**Roche**,**Germany**). The Human Insulin (INS) ELISA kit (**SunRed Biotechnology Company in Shanghi**,**China**) (Catalogue number: 201-12-0011) was used in the measurement of fasting insulin levels. The following equation was used to determine the homeostasis model assessment parameter of insulin resistance (HOMA-IR): [HOMA-IR = fasting insulin (U/mL) fasting plasma glucose (mg/dL)] /405. (HOMA-B): [20 × fasting insulin (µU/ml)]/ [fasting plasma glucose (mg/dl) − 63]. (HOMA S): [1/HOMA-IR] × 100%]^18^. The triglyceride–glucose index (TyG index) was measured using the formula: TyG index = Ln (fasting triglycerides [mg/dL] x fasting glucose [mg/dL])/2^19^.

#### Serum Asprosin and serum Gremlin-1 levels

A double-antibody sandwich ELISA technique is used by the Human Asprosin ELISA Kit from **SunRed Biotechnology Company in Shanghi**,** China** (catalogue No. 201-12-7691) and the Human Gremlin-1 ELISA Kit from the same company (catalogue No. 201-12-2736) to measure the levels of human serum Asprosin and serum Gremlin-1 in samples, respectively. The protocols provided with the kits were followed during the operations^15,16^.

The assay was performed in compliance with the manufacturer’s instructions. Each well received 50 µL of sample, different standards, or blank, were measured once. The plate was sealed with a sealer once each well had received 50 µl of the detection antibody. Plate was kept at 37 °C for an hour. After the incubation period, three rounds of aspiration and washing were performed on each well. For each well, 100 µL of the diluted detector was added. Each well was aspirated and given five cycles of washing after the plate was covered and allowed to sit at 37 °C for an hour. 100 µL of the substrate solution was added to each well, which was then covered and left to stand at room temperature in the dark for ten minutes. Each well was filled with 50 µL of the stop solution. Within 50 min, the optical density at 450 nm was determined. The Sunrise^™^ absorbance reader (**Tecan Trading AG**,** Männedorf**,** Switzerland**) produced a standard curve. This allowed the determination of the corresponding concentration for the sample OD value.

#### Peripheral blood asprosin gene and Gremlin-1 gene expression

*RNA extraction* Using an RNA extraction kit, all of the RNAs from the whole blood were isolated. The manufacturer’s instructions were followed for performing the RNA extraction (**GENEzolTM Reagent**,** Geneaid**,** Taiwan**) (catalogue number: GZR100). One ng of RNA was used for each reverse transcription procedure, and the quantity and quality of the RNA were both measured using the Nanodrop 2000 spectrophotometer (**Thermo Fisher**,** Waltham**,** MA**,** USA**).

*Reverse transcription* The cDNA was produced from it using the Top Script^™^ RT reverse transcription kit (**Enzynomics Inc.**,** Korea**) (catalogue number: RT 220), as directed by the manufacturer.

*Real-time PCR* The Asprosin and Gremlin-1 gene expression were determined using quantitative real-time PCR (RT-qPCR) using a StepOne^™^ System real-time PCR apparatus (**Applied Biosystems**,** Foster City**,** CA**,** USA**) and the TOPreal^™^ qPCR 2X Pre-MIX (SYBR Green with low ROX) kit (**Enzynomics Inc.**,** Korea**) (catalogue number: RT500s).

*Real-time PCR methodology* The reaction system was restricted to a volume of 20 µL. 10 µL of TOPreal^™^ qPCR 2X PreMIX, 1 µL of the forward primer, 1 µL of the reverse primer, 5 µL of the template cDNA, and 3 µL of PCR-high quality water were used to create a PCR reaction.

The RT-qPCR thermal process was programmed to operate for 15 min at 95 °C, then 40 cycles of 15 s at 95 °C, 30 s at 52 °C, and 30 s at 72 °C each. An analysis of the annealing curve was done to confirm the reaction’s specificity. Table 1 displays the primer sequence information. The mRNA expression of a known housekeeping gene, human GAPDH, was used to normalize the relative mRNA expression of the target genes. Comparing the results to the control group, fold alternations produced using the − 2^ΔΔCT^ technique are displayed^20^.

**Table 1:** Primers sequences.

Genes	Sequence (5’- 3’)	Accession No.
Asprosin	F: 5’-CGAATCCTAGAGCTCCTGCC-3’ R: 5’-GGAGGTAGCTGACCCCTTCT-3’	NM_001406716.1
Gremlin-1	F: 5’- CTGCTGAAGGGAAAAAGAA-3’ R: 5’-GATGGATATGCAACGACACT-3’	NM_001191323.2
GAPDH	F: 5’-CACCAGGGCTGCTTTTAACTC-3’ R: 5’-GACAAGCTTCCCGTTCTCAG-3’	NM_001357943.2

F: forward; R: reverse, GAPDH: Glyceraldehyde 3-phosphate dehydrogenase.

### Statistical analysis

The data were examined using the Shapiro-Wilk test, which revealed a non-parametric distribution. The Mann-Whitney, independent T-test, and the parameters were compared using chi-squared test. The strength of the correlation was assessed by means of the Spearman’s correlation test. The laboratory tests’ efficacy was assessed using a Receiver Operator Characteristic (ROC) curve. The 95% confidence interval (CI) and the area under the ROC curve (AUC) were calculated. To explain the risk factors, logistic regression analysis was used to compute the odds ratio (OR). Statistical significance was defined as a p-value less than 0.05. The statistical software that was used was SPSS 20 (SPSS Inc., Chicago, IL, USA)^21^.

## Results

### Demographic characteristic of studied groups

Table 2 lists the clinical parameters for all subjects. Age, BMI, and sex were comparable among the T1DM patients as well as the controls.

### Laboratory data of studied groups

As expected, patients with T1DM showed higher lipid profile abnormalities than controls, except for HDL-C and higher glycemic control markers (fasting glucose, HbA1c, and HOMA-IR). Fasting insulin showed lower values in T1DM patients in comparison to controls. No evident variations in HDL-C were seen between the two groups. Patients had higher TyG index in comparison with controls Table 2.

**Table 2:** Demographic and laboratory data of the studied groups.

	Type 1 diabetes group	Control group	*P*
	*N* = 50 (%)	*N* = 50 (%)	
Gender			
Female	18 (36%)	20 (40%)	0.771
Male	32 (64%)	30 (60%)	
Age (year)	9.24 ± 2.49	9.6 ± 2.65	0.622
BMI, (kg/m^2^)	20.6 ± 2.17	20.13 ± 1.9	0.38
Cholesterol, (mg/dL)	168 ± 51.51	127 ± 27.7	0.001**
Triglycerides, (mg/dL)	110 ± 22.17	88 ± 20.03	0.001**
HDL-C, (mg/dL)	40 ± 9.45	44 ± 10.54	0.204
LDL-C, (mg/dL)	105 ± 56.27	65 ± 26.58	0.003*
HbA1c, (%)	7.76 ± 1.39	4.98 ± 0.48	< 0.001**
Glucose, (mg/dL)	180.32 ± 33.13	80.82 ± 7.76	< 0.001**
Insulin (mU/L)	9.22 ± 1.43	11.93 ± 3.09	< 0.001**
HOMA IR	3.99[3.62–4.36]	2.37[2.03–2.73]	< 0.001**
HOMA-B	1.77 (1.32–2.07)	14.66 (10.07–20.16)	< 0.001**
HOMA-S	25.05 (22.95–27.63)	42.24 (36.66–49.25)	< 0.001**
TyG index	9.17 ± 0.24	8.15 ± 0.26	< 0.001*

Data represented as mean ± SD, Number (percentage), or median [IQR].

BMI: Body Mass Index, HDL-C: High density lipo-protein-cholesterol, LDL-C: Low density lipo-protein-cholesterol, HOMA-IR: Homeostatic model assessment for insulin resistance, HOMA-B: Homeostatic Model Assessment of Beta cell function, HOMA-S: Homeostatic Model Assessment of Insulin Sensitivity HbA_1_c: Hemoglobin A_1_c; TyG: triglyceride–glucose.

*p < 0.05 is statistically significant.

### Serum protein level and gene expression of Asprosin and Gremlin-1 results

As shown in Table 3, serum Asprosin concentration, serum Gremlin-1 concentration and their gene expression in the peripheral blood were significantly increased in the T1DM group in contrast to healthy individuals (*p* < 0.001).

**Table 3:** Serum protein level and gene expression of Asprosin and Gremlin-1 in different study groups.

Markers	Type 1 diabetes group *N* = 25	Control group *N* = 25	*P*
Serum protein level			
Serum Asprosin concentration, (ng/mL)	4.3 ± 1.49	2.45 ± 0.68	< 0.001*
Serum Gremlin-1 concentration, (ng/mL)	26.9 [15.65–36.7]	10.3 [6.9–14.45]	< 0.001*
Gene expression			
Asprosin expression, (Fold change)	3.421 ± 1.516	1.004 ± 0.093	< 0.001*
Gremlin-1 expression, (Fold change)	2.607 ± 0.811	1.001 ± 0.056	< 0.001*

Data represented as mean ± SD or median [IQR].

*Statistically significant.

### The receiver operation curve (ROC) study

**Fig. 1:**
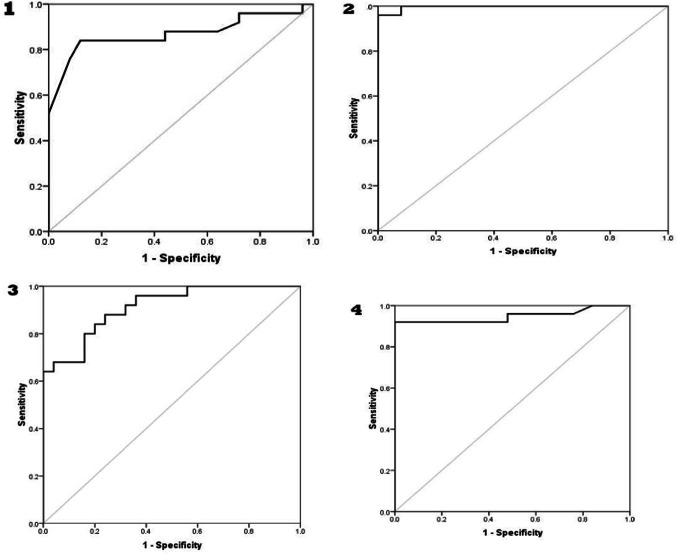
Figure 1 ROC curves that indicate performance of (**1**) serum Asprosin concentration, (**2**) Asprosin expression, (**3**) serum Gremlin-1 concentration, (**4**) Gremlin-1 expression in diagnosis of T1DM.

As demonstrated in Fig. 1, ROC curves of studied markers values for predicting the prevalence of T1DM have been built. AUC of serum Asprosin concentration, Asprosin expression, serum Gremlin-1 concentration, and Gremlin-1 expression were 0.87, 0.997, 0.912, and 0.949, respectively. The diagnostic performance criteria for T1DM prediction are presented in Table 4. Asprosin expression was the best marker for T1DM diagnosis with 96% sensitivity, 92% specificity, and 94% accuracy.

**Table 4:** Performance characteristics of serum Asprosin concentration, asprosin expression, serum Gremlin-1 concentration, and Gremlin-1 expression in diagnosis of type 1 diabetes.

Marker	Cutoff	AUC	Sensitivity	Specificity	PPV	NPV	Accuracy	*p*
Serum protein level								
Serum Asprosin concentration, (ng/mL)	≥ 3.35 ng/mL	0.87	84%	88%	87.5%	84.6%	86%	< 0.001*
Serum Gremlin-1 concentration, (ng/mL)	≥ 14.8 ng/mL	0.912	84%	80%	80.8%	83.3%	82%	< 0.001*
Gene expression								
Asprosin expression, (fold change)	≥ 1.156 fold change	0.997	96%	92%	92.3%	95.8%	94%	< 0.001*
Gremlin-1 expression, (fold change)	≥ 1.081 fold change	0.949	92%	88%	88.5%	91.7%	90%	< 0.001*

AUC: area under curve; PPV: positive predictive value; NPV: negative predictive value.

*p < 0.05 is statistically significant.

### Predictive value of cutoff of studied markers

The predictive value of the cutoff of serum Asprosin concentration, Asprosin expression, serum Gremlin-1 concentration and Gremlin-1 expression in the diagnosis of T1DM was assessed (Table 5). Asprosin expression can significantly increase the prevalence of T1DM by 276 folds, followed by Gremlin-1 expression, which can significantly increase the prevalence of T1DM by 84.33 folds. Serum Asprosin concentration and serum Gremlin-1 concentration can significantly increase the prevalence of T1DM by 38.5 and 21 folds, respectively (Table 5).

**Table 5:** Predictive value of cutoff of serum Asprosin concentration, asprosin expression, serum Gremlin-1 concentration and Gremlin-1 expression in diagnosis of T1DM.

Marker	Type 1 DM	Control group	COR (95% CI)	P
	*N* = 50(%)	*N* = 50(%)		
Serum protein level				
Serum Asprosin concentration ≥3.35 ng/mL	21(92%)	3(12%)	38.5[7.68–192.99]	< 0.001**
Serum Gremlin-1 concentration ≥14.8 ng/mL	21(84%)	5(20%)	21[4.92–89.56]	< 0.001**
Gene expression				
Asprosin expression ≥ 1.156 fold change	24(96%)	2(8%)	27[23.4–3255.32]	< 0.001**
Gremlin-1 expression ≥ 1.081fold change	23(92%)	3(12%)	84.33[12.84–553.92]	< 0.001**

COR: Crude odds ratio; CI: Confidence interval.

*p < 0.05 is statistically significant.

### Correlation analysis

Asprosin and Gremlin-1 (both serum protein level and their gene expression in the peripheral blood) demonstrated no discernible difference between genders (*P* > 0.05). According to what is stated in Table 6, Spearman correlation analysis revealed that serum Asprosin concentration and its gene expression were positively correlated with cholesterol, triglycerides, LDL-C, HbA1c, glucose, HOMA-IR, serum Gremlin-1 concentration, and Gremlin-1 expression. But a negative correlation was detected with insulin levels. The values of serum Gremlin-1 concentration and its gene expression were positively correlated with triglycerides, HbA1c, glucose, HOMA-IR, serum Gremlin-1 concentration, and Gremlin-1 expression (*P* < 0.001). But a negative correlation was detected with insulin levels. All studied markers were positively correlated with TyG index.

**Table 6:** Correlation between serum Asprosin concentration, asprosin expression, serum Gremlin-1 concentration, Gremlin-1 expression and the studied parameters.

Parameter	Serum protein Level				Gene expression			
	Asprosin		Gremlin-1		Asprosin expression		Gremlin-1 expression	
	*R*	*P*	*R*	*P*	*R*	*P*	*r*	*p*
Age, (year)	-0.021	0.884	-0.148	0.305	0.043	0.767	-0.024	0.869
BMI, (kg/m^2^)	0.189	0.189	-0.011	0.942	0.187	0.193	0.142	0.327
Cholesterol, (mg/dL)	0.389	0.008*	0.262	0.066	0.33	0.019*	0.257	0.071
Triglycerides, (mg/dL)	0.408	0.003*	0.411	0.003*	0.467	0.001*	0.329	0.019*
HDL-C, (mg/dL)	-0.222	0.122	-0.214	0.136	-0.187	0.194	0.011	0.939
LDL-C, (mg/dL)	0.359	0.01*	0.233	0.103	0.278	0.051	0.203	0.157
HbA1c, (%)	0.549	< 0.001*	0.601	< 0.001*	0.775	< 0.001*	0.708	< 0.001*
Glucose, (mg/dL)	0.583	< 0.001*	0.739	< 0.001*	0.765	< 0.001*	0.65	< 0.001*
Insulin, (mU/L)	-0.289	0.041*	-0.441	0.001*	-0.471	0.001*	-0.339	0.016*
HOMA IR	0.552	< 0.001*	0.643	< 0.001*	0.728	< 0.001*	0.666	< 0.001*
HOMA-β	-0.545	< 0.001**	-0.545	< 0.001**	-0.772	< 0.001**	-0.772	< 0.001**
HOMA-S	-0.552	< 0.001**	-0.552	< 0.001**	-0.728	< 0.001**	-0.728	< 0.001**
TyG index	0.554	< 0.001*	0.752	< 0.001*	0.696	< 0.001*	0.635	< 0.001*
Serum Asprosin concentration	1	–	0.468	0.001	0.565	< 0.001*	0.384	0.006*
Serum Gremlin 1concentration	0.468	< 0.001*	1	–	0.637	< 0.001*	0.467	0.001*
Asprosin expression	0.565	< 0.001*	0.637	< 0.001	1	–	0.723	< 0.001*
Gremlin-1 expression	0.384	0.006*	0.467	0.001	0.723	< 0.001*	1	–

BMI: Body Mass Index, HDL-C: High density lipo-protein-cholesterol, LDL-C: Low density lipo-protein- cholesterol, HOMA-IR: Homeostatic model assessment for insulin resistance, HbA_1_c: Hemoglobin A1c, r: Spearman rank correlation coefficient, TyG: triglyceride–glucose.

*p < 0.05 is statistically significant.

## Discussion

The etiology of T1DM, which affects people from various genetic and environmental backgrounds, has been intensely disputed for a long time^1^. Among the most common endocrine and metabolic diseases in youngsters is T1DM. Childhood-onset in addition to having negative effects on health care and resources, T1DM is a serious, disabling condition with life-threatening complications^1^.

As expected, there was a statistically significant difference in T1DM with regard to glycemic parameters. One possible explanation could be the lack or insufficient production of Insulin by beta cells in patients with T1DM. Insufficient insulin causes glucose to accumulate in the blood rather than enter cells^22^. In line with our findings, an earlier investigation discovered a connection between HbA1c and T1DM (*P* < 0.0001)^23^.

Asprosin has generated a lot of interest. it may be used as a therapeutic target and biomarker for metabolic diseases such obesity, diabetes, and polycystic ovarian syndrome (PCOS)^24^. Gremlin-1 has recently been found to inhibit the signaling of insulin in human skeletal muscle, liver, and adipocyte cells^15^. The activity of gremlin-1 is mediated by its interaction with BMPs or with membrane receptors such as the vascular endothelial growth factor receptor 2 (VEGFR2) or heparan sulfate proteoglycans (HSPGs). Gremlin-1 is involved in dysfunction during diabetes, obesity and non-alcoholic fatty liver disease (NAFLD) metabolic disorders^25^. The relevance of Asprosin and Gremlin-1 (serum protein level and its gene expression) in the identification of T1DM patients is still unclear, though. Because of this, one of the objectives of the current study was to investigate the relationship between these adipokine markers and metabolic changes in T1DM.

Our findings indicate that Asprosin exhibits statistically significant levels of both protein and gene expression in T1DM. This could be explained by the fact that Asprosin increases blood glucose release via the cAMP pathway when it binds to the olfactory receptor OLFR734. Additionally, it has been demonstrated that Asprosin reduces the amount of insulin secreted by inducing inflammation, dysfunctional beta cells, and death in pancreatic beta cells^26^. Our findings support a previous study’s finding that found a positive correlation between serum insulin concentrations and serum Asprosin levels during a fasting condition prior to the test^6^.

Based on our research, Gremlin-1 is expressed at statistically significant levels in T1DM at both the protein and gene levels. Our results supported a previous study that stated that diabetic individuals had higher levels of Gremlin-1 when compared to control subjects^27^.

We identified the cut-off values that most effectively distinguish between T1DM patients and control groups using ROC curves analysis. Our findings indicate that Asprosin gene expression is the best marker in the detection of T1DM, where it can considerably raise T1DM risk by a factor of 276. This was confirmed by an analysis of the predictive value of these markers in the diagnosis of T1DM was assessed. This is consistent with a number of clinical investigations that found that obese patients, T1DM, and type 2 diabetic mellitus (T2DM) have high level of serum Asprosin concentration^28^. Another study revealed that both T1DM and T2DM patients’ responses to glucose fluctuations appeared to be impaired while using Asprosin^24^.

The current study also demonstrated that Gremlin-1 expression and serum Gremlin-1 concentration can considerably raise T1DM risk by 84.33 and 21 times, respectively. This validates another study’s main result that persons with T1DM and diabetic kidney disease (DKD) have significantly higher urine levels of Gremlin-1^29^. In a study on animals, mice without the Gremlin-1 gene were protected from the artificially created T1DM model^27^. These findings suggest serum Gremlin-1 concentration may serve as a biomarker for the prediction of T1DM^30^.

In this study, there was no statistically significant difference between males and females in Asprosin and Gremlin-1 (both their serum protein level and their gene expression in peripheral blood). These results can be explained by three facts: first, females have more adipose tissue than males do, but males are more prone to develop visceral WAT. There is no gender-based differentiation, then. Second, Asprosin secretion from WAT is glucogenic and orexigenic and depends on fasting. Third, while the majority of this multipurpose adipokine comes from WAT, other tissues such cartilage, pancreatic B-cells, and salivary glands may also generate Asprosin^24^. Our results confirm the results of Clemente-Suárez et al.^31^ examined the differences in serum Asprosin concentration between males and females and discovered none. According to previous study, there are gender differences in how Asprosin affects pediatric obesity. This study found that fat males had much lower Asprosin serum concentrations than fat females did^31^. Serum Asprosin concentration and female gender were found to be positively and linearly correlated by previous study^32^. According to a recent study, changes in serum Asprosin concentration did not correspond with HbA1c, age, or gender^6^.

Furthermore, no marker showed an age correlation. Although adipose tissue increases with middle age, there was no correlation because study participants were all in the same age group. This is consistent with prior study showing no relationship between age and Asprosin^31^.

Additionally, based on our findings, there is no statistically significant link between Asprosin and Gremlin-1 (gene expression and serum protein level) and BMI. The explanation is that there is a discrepancy between the amount of adipokine in the tissue and the amount released into the bloodstream^5^. Another possibility is that, despite being primarily produced by adipose tissue, adipokines can also be secreted by other tissues^16^. Therefore, there may be less correlation between the levels of these markers and the indications of obesity. Our findings contradict earlier study that found a link between Asprosin and BMI^31^.

One finding of the current study is that lipid profile factors like cholesterol and triglycerides significantly correlated with Asprosin (both protein level and gene expression). One possible explanation is that free fatty acids, which are discharged into the bloodstream to support muscular performance, are produced through the hydrolysis of triacylglycerols, an energy metabolite found in adipose tissue. Participants who are overweight or obese experience a significant decrease in serum Asprosin concentration following moderate intensity aerobic exercises^33^. This backs up the findings of Groener et al.^6^, who found that the expected increase in serum Asprosin concentration was not reflected in the clamp test results during hypoglycemia in patients with greater LDL-C and lower HDL-C levels. Therefore, it appears that these characteristics are related to variations in serum Asprosin concentration or Asprosin release in addition to circulating Asprosin levels. The TyG index can predict diabetes, measure poor glycemic management, and evaluate pancreatic β cell activity. The TyG index was found to be significantly associated with cardiometabolic risk, vascular diseases, and cardiovascular events^19^. The studied markers were positively correlated with that index which requires further evaluation of these markers as predictors for cardiovascular events.

Serum Asprosin concentrations were considerably higher in obese children compared to lean controls. Children with insulin resistance (IR) had greater Asprosin levels compared to the non-IR group. Asprosin correlated positively with the leptin-to-adiponectin ratio^34^.

One finding of the current study is that glycemic parameters like HbA1c, glucose, and HOMA IR significantly correlated with Gremlin-1 (both protein level and gene expression). This might be because Gremlin-1 has a negative correlation with the Insulinogenic Index (a measure of beta cell insulin production). Thus, as Gremlin-1 levels rise, the Insulinogenic Index falls, which thus lowers the risk of insulin insufficiency (T1DM)^27^. This is in line with a prior study that found a favorable correlation between insulin levels and Gremlin-1^15^.

Another finding of this study is that is that lipid profile factors like cholesterol and triglycerides significantly correlated with Gremlin-1 (both protein level and gene expression). The fact that recombinant Gremlin-1 increased lipogenesis, lipid accumulation, and endoplasmic reticulum stress indicators could help to explain this^35^. This is consistent with a previous study that found a favorable correlation between cholesterol and Gremlin-1^9^. Adiponectin serum concentrations were significantly higher in children with T1DM than in the control group^36^. A correlation between adiponectin levels was seen with adipose tissue Gremlin 1 mRNA levels^15^.

The authors are aware of certain limitations. The study’s population is primarily Egyptian, so it must first be carefully expanded to include other countries. It is recommended that cut-off points, which were determined using a small sample, be thoroughly examined and validated in a more extensive population. Third, the range for the duration of diabetes was extremely small. As a result, the majority of the study’s participants had just developed diabetes. Fourth, this study ignores other difficulties like atherosclerosis, cardiovascular problems, and renal impairment that may alter the release of adipokines. Furthermore, because this study was observational, causality cannot be shown. Some factors that affect Asprosin/Gremlin-1, such as physical activity, and diet, which affects Asprosin/Gremlin-1 production, were missing, even though the inadequate information about drugs and complications in the T1DM patients could contribute to the explanation of their varying levels. Also, the serum Asprosin could serve as a potential biomarker for predicting thyroid dysfunction in diabetes^37^. Unfortunately, the studied markers levels were not evaluated in T1DM associated thyroid dysfunction. Future studies on Asprosin and Gremlin-1 should give careful consideration to these limitations.

We recommend more studies to validate the significance of Asprosin and Gremlin-1 (both blood protein levels and their gene expressions) in the diagnosis of T1DM; more individuals are needed, to determine how diabetic treatment affects these markers and to investigate the correlation between these markers in type 1 diabetic patients and various diabetes complications.

Also, we recommend performing a comparison of these markers with other adipokines, such as adiponectin and leptin, which have a valuable effect on diabetes.

## Conclusions

In comparison to controls, T1DM patients had significantly greater levels of Asprosin and Gremlin-1 (both serum protein leveland their gene expressions). These indicators and the lipid and glycemic profiles seemed to be linked. Asprosin expression appeared to be the most effective marker for identifying T1DM. Apparently, Asprosin expression is a T1DM predictor.

## Data Availability

The data that support the findings of this study are available on request from the corresponding author.

## References

[1] Addissouky, T. A., Ali, M. M. A., El Sayed, I. E. T. & Wang, Y. Type 1 diabetes mellitus: retrospect and prospect. *Bull. Natl. Res. Centre*. **48**, 42. 10.1186/s42269-024-01197-z (2024). https://bnrc.springeropen.com/articles/

[2] Abouzid, M. R., Ali, K., Elkhawas, I. & Elshafei, S. M. An overview of diabetes mellitus in Egypt and the significance of integrating preventive cardiology in diabetes management. *Cureus* **14**, e27066 (2022). https://www.ncbi.nlm.nih.gov/pmc/articles/PMC9390800/36000101 10.7759/cureus.27066 PMC9390800

[3] Ley, S. H., Hamdy, O., Mohan, V. & Hu, F. B. Prevention and management of type 2 diabetes: dietary components and nutritional strategies. *Lancet* **383** (9933), 1999–2007 (2014). https://pubmed.ncbi.nlm.nih.gov/24910231/24910231 10.1016/S0140-6736(14)60613-9 PMC4751088

[4] Cade, W. T. Diabetes-related microvascular and macrovascular diseases in the physical therapy setting. *Phys. Ther.* **88**, 1322–1335 (2008). https://pmc.ncbi.nlm.nih.gov/articles/PMC2579903/#:~:text=Diabetes%20is%20a%20disease%20that,organ%20and%20tissue%20damage%20in. 10.2522/ptj.20080008 PMC2579903 18801863

[5] Axelsson, J., Heimbürger, O., Lindholm, B. & Stenvinkel, P. Adipose tissue and its relation to inflammation: the role of adipokines. *J. Ren. Nutr.* **15**, 131–136 (2005). https://pubmed.ncbi.nlm.nih.gov/15648022/15648022 10.1053/j.jrn.2004.09.034

[6] Groener, J. B. et al. Asprosin response in hypoglycemia is not related to hypoglycemia unawareness but rather to insulin resistance in type 1 diabetes. *PloS one*. 14, e0222771. (2019). https://www.ncbi.nlm.nih.gov/pmc/articles/PMC6752946/10.1371/journal.pone.0222771 PMC6752946 31536600

[7] Christou, M. A. et al. Effects of hypoglycemia on cardiovascular function in patients with diabetes. *International Journal of Molecular Sciences* 24 (11), 9357 (2023). https://www.mdpi.com/1422-0067/24/11/9357 10.3390/ijms24119357 PMC10253702 37298308

[8] Zhang, L. et al. Circulating asprosin concentrations are increased in type 2 diabetes mellitus and independently associated with fasting glucose and triglyceride. *Clin. Chim. Acta*. **489**, 183–188 (2019). https://pubmed.ncbi.nlm.nih.gov/29104036/29104036 10.1016/j.cca.2017.10.034

[9] Alregaiey, K. A. et al. Analysis of Gremlin 1 levels following Sleeve Gastrectomy. *Cureus* **15**, (2023). https://www.ncbi.nlm.nih.gov/pmc/articles/PMC10642626/10.7759/cureus.48738 PMC10642626 37965235

[10] Toschi, E. & Munshi, M. N. Benefits and challenges of Diabetes Technology Use in older adults. *Endocrinol. Metab. Clin. North. Am.* **49** (1), 57–67 (2020). https://pubmed.ncbi.nlm.nih.gov/31980121/31980121 10.1016/j.ecl.2019.10.001 PMC6983469

[11] Warshauer, J. T., Bluestone, J. A. & Anderson, M. S. New frontiers in the treatment of type 1 diabetes. *Cell Metabol.* **31** (1), 46–61 (2020). https://pubmed.ncbi.nlm.nih.gov/31839487/10.1016/j.cmet.2019.11.017 PMC6986815 31839487

[12] Nagy, G. et al. New therapeutic approaches for type 1 diabetes: disease-modifying therapies. *World J. Diabetes*. **13** (10), 835–850. 10.4239/wjd.v13.i10.835 (2022). 36312000 10.4239/wjd.v13.i10.835 PMC9606789

[13] Romere, C. et al. Asprosin, a Fasting-Induced glucogenic protein hormone. *Cell* **165**, 566–579 (2016). https://pubmed.ncbi.nlm.nih.gov/27087445/27087445 10.1016/j.cell.2016.02.063 PMC4852710

[14] Mishra, I. et al. Asprosin-neutralizing antibodies as a treatment for metabolic syndrome. eLife. 10, e63784.(2021). https://pubmed.ncbi.nlm.nih.gov/33904407/10.7554/eLife.63784 PMC8102062 33904407

[15] Hedjazifar, S. et al. The novel adipokine Gremlin 1 antagonizes insulin action and is increased in type 2 diabetes and NAFLD/NASH. *Diabetes* **69**, 331–341 (2020). https://pubmed.ncbi.nlm.nih.gov/31882566/31882566 10.2337/db19-0701

[16] Algul, S. et al. Evaluation of Aerobic Exercise Induced metabolic stress on serum asprosin levels: comparison of Fitness Status. *PROGRESS Nutr.* **23**, (2021). https://mattioli1885journals.com/index.php/progressinnutrition/article/view/11579

[17] American Diabetes Association. *Standards of Medical Care in Diabetes—2020* abridged for primary care providers. *Clin. Diabetes*. **1** (1), 10–38. 10.2337/cd20-as01 (January 2020). 10.2337/cd20-as01 PMC6969656 31975748

[18] Fujii, H. et al. HOMA-IR: an independent predictor of advanced liver fibrosis in nondiabetic non‐alcoholic fatty liver disease. *J. Gastroenterol. Hepatol.* **34**, 1390–1395 (2019). https://pubmed.ncbi.nlm.nih.gov/30600551/30600551 10.1111/jgh.14595

[19] Kurniawan, L. B. Triglyceride-glucose index as a biomarker of insulin resistance, diabetes Mellitus, metabolic syndrome, and Cardiovascular Disease. *Rev. EJIFCC*. **35** (1), 44–51 (2024). https://pubmed.ncbi.nlm.nih.gov/38706737/PMC11063788 38706737

[20] Livak, K. J. & Thomas, D. S. Analysis of relative gene expression data using real-time quantitative PCR and the 2 – ∆∆CT method. *Methods* **25**, 402–408 (2001). https://pubmed.ncbi.nlm.nih.gov/11846609/11846609 10.1006/meth.2001.1262

[21] Kovács, D. et al. Sebocytes differentially express and secrete adipokines. *Exp. Dermatol.* **25**, 194–199 (2016). https://pubmed.ncbi.nlm.nih.gov/26476096/26476096 10.1111/exd.12879

[22] Rodrigues, O. et al. Type 1 diabetes mellitus: a review on advances and challenges in creating insulin producing devices. *Micromachines* **14**, 151 (2023). https://pubmed.ncbi.nlm.nih.gov/36677212/36677212 10.3390/mi14010151 PMC9867263

[23] Strollo, R. et al. Autoantibody and T cell responses to oxidative post-translationally modified insulin neoantigenic peptides in type 1 diabetes. *Diabetologia* **66**, 132–146 (2023). https://pubmed.ncbi.nlm.nih.gov/36207582/36207582 10.1007/s00125-022-05812-4 PMC9729141

[24] Farrag, M. et al. Asprosin in health and disease, a new glucose sensor with central and peripheral metabolic effects. Frontiers in Endocrinology. 13, 1101091 (2023). https://www.ncbi.nlm.nih.gov/pmc/10.3389/fendo.2022.1101091 PMC9849689 36686442

[25] Grillo, E., Ravelli, C., Colleluori, G., D’Agostino, F., Domenichini, M., Giordano, A., et al. Role of gremlin-1 in the pathophysiology of the adipose tissues. Cytokine Growth Factor Revi. 69, 51–60. (2023). https://pubmed.ncbi.nlm.nih.gov/36155165/10.1016/j.cytogfr.2022.09.004 36155165

[26] Shabir, K. et al. Asprosin exerts pro-inflammatory effects in THP-1 macrophages mediated via the toll-like receptor 4 (TLR4) pathway. *Int. J. Mol. Sci.* **24**, 227 (2022). https://www.ncbi.nlm.nih.gov/pmc/articles/PMC9820073/36613673 10.3390/ijms24010227 PMC9820073

[27] Al-Regaiey, K. A. et al. Relationship of plasma Gremlin 1 levels with body adiposity and glycemic control in Saudi female type 2 diabetes patients. *Diabetes Metabolic Syndrome Obesity: Targets Therapy* 3429–3436. (2022). https://pubmed.ncbi.nlm.nih.gov/36353668/10.2147/DMSO.S372146 PMC9639591 36353668

[28] Xu, L. et al. Association between serum asprosin and diabetic nephropathy in patients with type 2 diabetes mellitus in the community: a cross-sectional study. *Diabetes Metabolic Syndrome Obesity: Targets Therapy*. **15**, 1877–1884 (2022). https://www.ncbi.nlm.nih.gov/pmc/articles/PMC9215350/35757196 10.2147/DMSO.S361808 PMC9215350

[29] Afkarian, M. et al. Urinary excretion of RAS, BMP, and WNT pathway components in diabetic kidney disease. *Physiological reports.* 2, e12010 (2014). https://www.ncbi.nlm.nih.gov/pmc/articles/PMC4098738/10.14814/phy2.12010 PMC4098738 24793984

[30] McKnight, A. J. et al. A GREM1 gene variant associates with diabetic nephropathy. *J. Am. Soc. Nephrol.* **21**, 773–781 (2010). https://pubmed.ncbi.nlm.nih.gov/20150533/20150533 10.1681/ASN.2009070773 PMC2865734

[31] Clemente-Suárez, V. J. et al. The role of adipokines in health and disease. *Biomedicines* **11**, 1290 (2023). https://pubmed.ncbi.nlm.nih.gov/37238961/37238961 10.3390/biomedicines11051290 PMC10216288

[32] Mirr, M. et al. Serum asprosin correlates with indirect insulin resistance indices. *Biomedicines* **11**, 1568 (2023). https://pubmed.ncbi.nlm.nih.gov/37371663/37371663 10.3390/biomedicines11061568 PMC10295799

[33] Huang, R. et al. A cross-sectional comparative study on the effects of body mass index and exercise/sedentary on serum asprosin in male college students. *Plos One*. **17**, e0265645 (2022). https://www.ncbi.nlm.nih.gov/pmc/articles/PMC8982887/#:~:text=The%20study%20found%20that%20when,a%20greater%20decline%20%5B16%5D 35381008 10.1371/journal.pone.0265645 PMC8982887

[34] Wang, M. et al. Serum asprosin concentrations are increased and Associated with insulin resistance in children with obesity. *Ann. Nutr. Metab.* **75** (4), 205–212. 10.1159/000503808 (2019). 31775140 10.1159/000503808

[35] Choi, S. W. et al. Adipokine gremlin-1 promotes hepatic steatosis via upregulation of ER stress by suppressing autophagy‐mediated signaling. *J. Cell. Physiol.* **238**, 966–975 (2023). https://pubmed.ncbi.nlm.nih.gov/36890751/36890751 10.1002/jcp.30982

[36] Majewska, K. A., Majewski, D., Skowrońska, B., Stankiewicz, W. & Fichna, P. Serum leptin and adiponectin levels in children with type 1 diabetes mellitus - relation to body fat mass and disease course. *Adv. Med. Sci.* **61** (1), 117–122. 10.1016/j.advms.2015.10.002 (2016). 26647091 10.1016/j.advms.2015.10.002

[37] Lefta, N. A., Abed, A. Y. & Abed, B. A. Estimation of Asprosin Levels in female Iraqi patients with type 2 diabetes and hypothyroidism. *J. Med. Chem. Sci.* **6** (2), 433–439. 10.26655/JMCHEMSCI.2023.2.23 (2023).

